# Stimulation of glioma cell motility by expression, proteolysis, and release of the L1 neural cell recognition molecule

**DOI:** 10.1186/1475-2867-9-27

**Published:** 2009-10-29

**Authors:** Muhua Yang, Shalini Adla, Murali K Temburni, Vivek P Patel, Errin L Lagow, Owen A Brady, Jing Tian, Magdy I Boulos, Deni S Galileo

**Affiliations:** 1 Department of Biological Sciences, University of Delaware, Newark, DE 19716 USA; 2 Helen F. Graham Cancer Center, Christiana Care Health System, Newark, DE 19713 USA; 3 Department of Biology, Georgian Court University, Lakewood, NJ 08701 USA

## Abstract

**Background:**

Malignant glioma cells are particularly motile and can travel diffusely through the brain parenchyma, apparently without following anatomical structures to guide their migration. The neural adhesion/recognition protein L1 (L1CAM; CD171) has been implicated in contributing to stimulation of motility and metastasis of several non-neural cancer types. We explored the expression and function of L1 protein as a stimulator of glioma cell motility using human high-grade glioma surgical specimens and established rat and human glioma cell lines.

**Results:**

L1 protein expression was found in 17 out of 18 human high-grade glioma surgical specimens by western blotting. L1 mRNA was found to be present in human U-87/LacZ and rat C6 and 9L glioma cell lines. The glioma cell lines were negative for surface full length L1 by flow cytometry and high resolution immunocytochemistry of live cells. However, fixed and permeablized cells exhibited positive staining as numerous intracellular puncta. Western blots of cell line extracts revealed L1 proteolysis into a large soluble ectodomain (~180 kDa) and a smaller transmembrane proteolytic fragment (~32 kDa). Exosomal vesicles released by the glioma cell lines were purified and contained both full-length L1 and the proteolyzed transmembrane fragment. Glioma cell lines expressed L1-binding αvβ5 integrin cell surface receptors. Quantitative time-lapse analyses showed that motility was reduced significantly in glioma cell lines by 1) infection with an antisense-L1 retroviral vector and 2) L1 ectodomain-binding antibodies.

**Conclusion:**

Our novel results support a model of autocrine/paracrine stimulation of cell motility in glioma cells by a cleaved L1 ectodomain and/or released exosomal vesicles containing L1. This mechanism could explain the diffuse migratory behavior of high-grade glioma cancer cells within the brain.

## Background

Malignant gliomas are brain cancers that are particularly insidious because of their extremely invasive behavior and are lethal mostly because of their rapid spreading in the brain [1,2]. They often follow existing anatomical structures such as nerve fiber tracts and blood vessels, but glioma cells also can migrate diffusely through the neuropil (i.e. diffuse glioma). Diffuse migration as single cells or small groups is a primary reason for therapeutic failure, and there are no imaging techniques available that can detect such diffuse migration. The mechanisms by which glioma cell invasion occur are poorly understood. Recently, many researchers have uncovered alterations in gene expression at different stages of malignant transformation of these cells. It is now important to identify which of these or other changes are related to glioma invasiveness and to what extent experimental alterations in expression of specific genes govern this important feature [3]. The neural cell adhesion/recognition protein L1 (L1CAM; CD171) is abnormally expressed in multiple cancer cell types, including high-grade gliomas [4]. Whereas L1 is not expressed in normal tissues from which the cancer cells arise [5], it has been implicated in motility and metastasis in some malignant cancers [6,7].

L1 is a 200-220 kDa type I membrane glycoprotein belonging to the immunoglobulin superfamily that contains six immunoglobulin-like domains (Ig domains), five fibronectin-like repeats (FN repeats), a transmembrane domain, and a highly conserved cytoplasmic domain [8,9]. L1 has homophilic (L1-L1) binding via several Ig domains [10] and also has heterophilic interactions with axonin-1 [11], CD24 [12], the proteoglycan neurocan and several integrins [13]. The significance of L1's function in central nervous system development is well established, which includes facilitating neuronal migration, neuronal survival, as well as axon outgrowth, guidance, fasciculation and regeneration, [14-17]. Mutations in the human L1 gene cause a myriad of nervous system birth defects [18] such as Hydrocephalus due to congenital Stenosis of Aqueduct of Sylvius (HSAS) and MASA Syndrome. Although L1 was initially described in the nervous system and thought to be restricted to post-mitotic neurons in the adult central nervous system and pre- and non-myelinating Schwann cells in the adult peripheral nervous system [19,20], L1 expression is also found in hematopoietic and some epithelial cells [21-24].

L1 has been demonstrated to be expressed in different human cancers including lung cancer, glioma, melanoma, renal carcinoma, and colon carcinoma [4,25-31]. Furthermore, L1 is suggested to be a new tumor marker in those malignancies [5,32]. The expression of L1 in a variety of cancer types suggests the potential role of L1 in tumor cell adhesion and/or migration.

L1 is proteolyzed and released from the cell membrane by ADAM10 and ADAM17, two members of the disintegrin and metalloprotease (ADAM) family [33-37]. The soluble L1 ectodomain, after ADAM mediated proteolysis, has been suggested to interact with integrins to stimulate cell motility and cell migration [33]. The Arg-Gly-Asp (RGD) motif in the 6^th ^Ig domain of L1 interacts with several integrins including α5β1, αvβ3, αvβ5, αvβ1 as well as the platelet integrin αIIbβ3 [38,22,40,13,33]. The soluble L1 ectodomain has also been detected in serum samples of patients with melanoma, ovarian and uterine tumors and is suggested to increase cell migration and metastasis in those cancers [28,41]. L1 and ADAM10 mediated L1 proteolysis also induces metastasis in human colon cancer cells [37].

L1 is also released from melanoma and ovarian cancer cells extracellularly in the form of exosomes and can be cleaved by ADAMs within the exosomes [42,43]. Exosomes are minute membrane vesicles that are released from a variety of cell types including tumor cells, red blood cells, platelets, lymphocytes, and dendritic cells [43,44] via the fusion of multivesicular endosomes with the plasma membrane [45]. This suggests that exosomes might be another source of "soluble" L1 ectodomain from cancer cells in addition to that which occurs from ADAM proteolysis.

Although the expression of L1 and its potential stimulatory function in glioma motility has been approached [4,46-48], precise measurements of L1-mediated stimulation have not been documented. Here, we hypothesized that the soluble L1 ectodomain resulting from ADAM proteolysis and/or L1-containing exosomes stimulate glioma cell motility through integrins in an autocrine matter. We demonstrated that gliomas express and cleave L1, release exosomes containing L1, express receptors for L1, and glioma cell motility is stimulated by L1.

## Results

### L1 is expressed in high-grade glioma surgical samples

**Figure 1:**
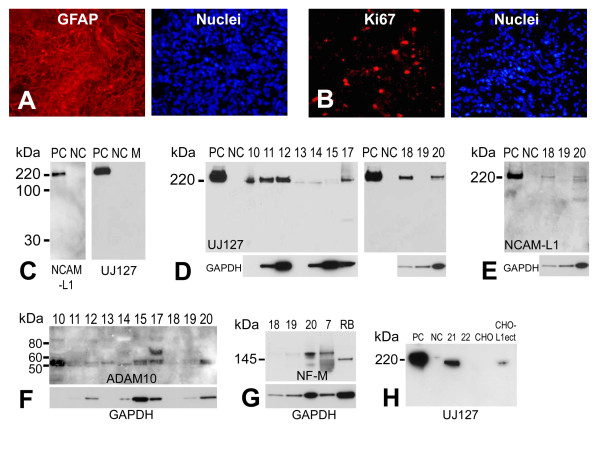
**Characterization of human primary glioma surgical samples**. Extensive GFAP (A, left panel) expression was detected in primary glioma surgical sample frozen sections using immunofluorescent staining with a rabbit anti-GFAP antibody. (B, left panel) Numerous dividing cells were detected in the same surgical glioma sample using an anti-Ki67 antibody. Right panels in (A) and (B) are nuclear counterstaining for GFAP and Ki67 stained sections, respectively. (C) Demonstration of specificity of anti-L1 antibodies anti-cytoplasmic polyclonal NCAM-L1 (left panel) and anti-ectodomain monoclonal UJ127 (right panel) by western blot analysis. Human L1-expressing quail QT6 cells (QT6/hFL1) were used as positive controls (PC), untransfected QT6 cells were used as negative controls (NC), and plain QT6 cell culture media (M) was used as an additional negative control. (D) L1 expression was found in human primary gliomas surgical samples (sample numbers 10-15 and 17-20) by western blot analysis using UJ127 anti-L1 antibody. Transfected QT6/hFL1 cells were used as positive control and QT6 cells were used as a negative control. Gels were loaded with 10 μg total protein and probed for GAPDH as a loading control (see text). (E) Analysis of surgical samples 18-20 using anti-L1 antibody NCAM-L1. Same blot was used as for (D, right panel, 10 μg total protein/well). GAPDH was used as a loading control. (F) Analysis of glioma surgical samples for L1 protease ADAM10, revealing that all samples were positive predominantly for active ADAM10 (approx. 55 kDa). (G) Surgical samples # 7, 18, 19, and 20 were analyzed by western blot with a rabbit anti-NF-M antibody to detect neurofilament expression. Adult rat brain (RB) lysate was used as a positive control for NF-M staining. (H) Media from surgical sample cells grown in culture were analyzed by western blot for L1. Soluble L1 was detectable in media from sample # 21. Positive controls (PC) were cell lysates from QT6/hFL1 cells, and untransfected cell lysate was used as negative control (NC). Media from CHO cells transfected with an L1 ectodomain vector (CHO-L1ecto) were also used as a positive control for soluble L1. Media from untransfected CHO cells were used as a negative control.

Eighteen high-grade (III-IV) glioma surgical samples were obtained as described in the methods. Such gliomas express glial fibrillary acidic protein (GFAP) [49-51] and are associated with high proliferation potential [52]. Here, the glioma surgical samples were stained with GFAP and Ki67 (a proliferation marker) to further confirm the identity and malignancy of the samples collected (Fig. 1A, B). L1 expression was assessed in these samples in two ways. Fourteen of the 18 surgical samples were directly lysed in RIPA lysis buffer, followed by electrophoresis and western blotting for L1 (Fig. 1C-E). Alternatively, cell cultures were made from dissociated surgical samples and kept in culture for 2, 4, or 15 weeks, followed by dissociation, protein extraction, and western blotting (4 of the 18 samples, data not shown). Seventeen of the 18 surgical samples showed L1 expression regardless of whether they were a fresh surgical sample or in culture for 2-15 weeks. L1 expression was variable (Fig. 1D; 10 μg/well), ranging from high-level to barely detectable and was only non-detectable in one sample (#19). The samples showed L1 expression by using either NCAM-L1 C-20 or UJ127 antibodies as a high molecular weight form of approximately 220 kDa. A 32 kDa membrane bound proteolytic fragment (the result of ADAM10 cleavage) was not found using the NCAM-L1 antibody (Fig. 1E), specific for a cytoplasmic epitope of L1. This suggests that L1 has not been proteolyzed in our surgical specimens, a phenomenon which does occur in the surgical sample cells in culture and in glioma cell lines (see below). Alternatively, the cleavage that might occur in the surgical specimens may occur at a level too low to visualize, or the 32 kDa cytoplasmic fragment might be cleaved by gamma-secretase [35]. Nevertheless, our results demonstrate L1 expression in the vast majority of our high-grade glioma samples and, thus, L1 may play a functional role in these tumors.

We analyzed surgical samples for L1 protease ADAM10 [31,33] by western blotting to determine if the reason that L1 was not proteolyzed in these samples was due to the absence of the protease. ADAM10 was expressed in all samples analyzed and primarily in its active lower molecular weight form (Fig. 1F, approximately 55 kDa band), even in sample 19 where L1 expression was not detected. The higher molecular weight band present in sample #17 most likely represents the inactive ADAM10 precursor. Thus, the lack of L1 proteolysis in surgical samples must be due to something other than the lack of ADAM10 expression.

To analyze for the potential presence of neurons that might contribute to the L1 that was detected above, a neuronal marker was assessed in four surgical specimens by western blotting. The middle weight neurofilament protein, NF-M, was detected in two specimens but was not detected or barely detected in two others (Fig. 1G). This protein normally is expressed in brain only in post mitotic neurons, which might suggest the presence of some neurons in 2 of the surgical samples. However, differentiated neuronal marker expression has been reported in GBMs previously [53-55] and NF proteins can be expressed in non-neuronal brain tumors [56]. Thus, we do not know which of the above possibilities occurred, but the NF-M expression could be by glioma cells themselves in the samples. Nonetheless, any neuronal inclusion within our glioma surgical samples is likely scant, at best, based on the abundance of GFAP staining (Fig. 1A).

Because L1 proteolysis was not apparent in fresh surgical samples (Fig. 1E), two samples were dissociated and placed in culture. Western blot analysis of cell culture supernatant using the anti-ectodomain antibody (UJ127) clearly showed the presence of the ectodomain fragment from sample # 21 (Fig. 1H). The soluble L1 from these cells was slightly lower in molecular weight than the cell surface L1 from QT6 cells transfected with a human L1 expression plasmid. Thus, some surgical high-grade glioma cells are able to proteolyze and release the L1 ectodomain, at least in culture.

### L1 in glioma cell lines is proteolyzed

**Figure 2:**
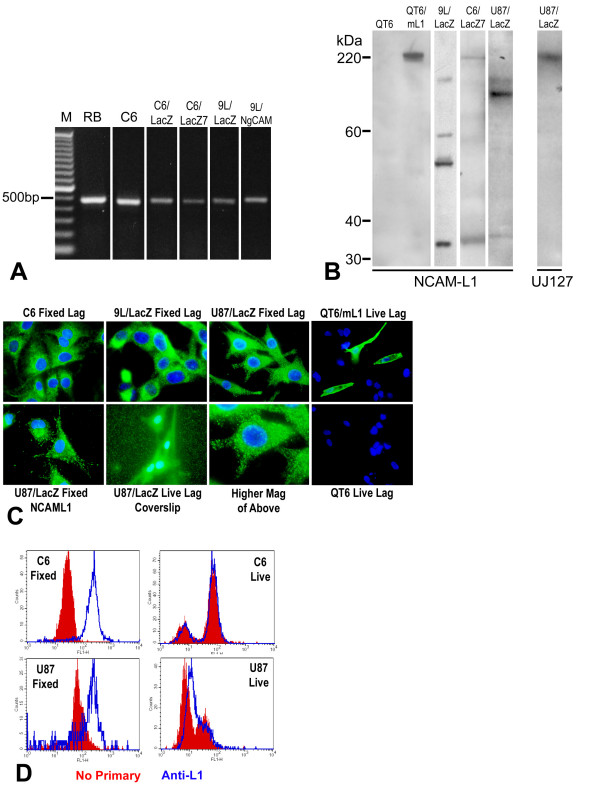
**L1 is expressed and proteolyzed in glioma cell lines**. (A) RT-PCR was performed on glioma cell lines to detect L1 mRNA. PCR primers were designed as described to generate a 461 bp L1 PCR product. Rat glioma cell lines C6, C6/LacZ, C6/LacZ7, 9L/NgCAM, 9L/LacZ exhibited the 461 bp band after RT-PCR. Rat brain (RB) served as a positive control for L1 mRNA expression. No-reverse transcriptase control reactions were run for every sample, and bands were not present in those lanes (not shown). (B) Western blot analysis of L1 expression. NCAM-L1 antibody (left panel) recognized L1 in rat glioma cell line 9L/LacZ, C6/LacZ7, and human glioma U87/LacZ cells and resulted in lower than full length bands. The approximate weight 32 kDa band indicates proteolysis by ADAM10 in all 3 cell lines. Further proteolysis in 9L/LacZ and U-87/LacZ cells is indicated by additional bands of lower than 200 kDa. Plain quail QT6 and mouse L1-expressing QT6 cells were used as negative and positive controls, respectively. Antibody UJ127 (right panel) against human L1 ectodomain was used for analysis of L1 in U87/LacZ human glioma cell line to determine if the complete ADAM10 ectodomain cleavage product was present, and the large fragment (approx. 220 kDa) indicates its presence. (C) Rat glioma cell lines C6, 9L/LacZ and the human glioma cell line U87/LacZ were immunostained on coverslips with anti-L1 antibodies. Lagenaur polyclonal and NCAM-L1 revealed bright punctate intracellular staining for L1 in fixed and permeablized cells in all 3 cell lines but surface staining was not detectable. Small punctate staining could be visualized on the level of the coverslip outside the cell boundaries when U87/LacZ cells were stained live using Lagenaur (lower middle-left panel). The lower middle-right panel is a higher magnification of upper right panel. The two rightmost panels show quail QT6 transfected with mouse L1 cDNA (top right) or untransfected QT6 (bottom right) immunostained live with the Lagenaur polyclonal antibody, with 3 bright surface stained transfected cells (top right). **(**D) FACS analyses of fixed (left two panels) and live (right two panels) cells. Fixed and permeablized cells exhibited distinct positive peaks of L1 immunofluorescence compared to no-primary control cells. However, live cells exhibited nearly identical fluorescence profiles to the no-primary control cells indicating little, if any, cell surface L1.

L1 expression and L1 proteolysis in glioma cell lines were tested using reverse transcription-polymerase chain reaction (RT-PCR), western blot, immunofluorescent staining, and flow cytometry (FACS) analysis. RT-PCR was used with primers spanning the transmembrane region and detected L1 mRNA in variants of rat C6 and 9L glioma lines including C6, C6/LacZ, C6/LacZ7, 9L/LacZ, and 9L/NgCAM cell lines (Fig. 2A). Rat glioma cell lines C6, C6/LacZ, C6/LacZ7, 9L/NgCAM, 9L/LacZ exhibited the 461 bp band after RT-PCR. Rat brain (RB) served as a positive control for L1 mRNA expression.

Western blot analysis was used to confirm L1 protein expression in two of the above rat glioma cell lines and a human glioma line U-87/LacZ (Fig. 2B). This analysis demonstrated proteolysis in all three cell lines analyzed, however, the patterns of proteolysis differed in each cell line. In C6/LacZ7 cells the NCAM-L1 antibody detected the 220 kDa surface full length L1, but 9L/LacZ and U-87/LacZ cells exhibited high molecular weight bands that were smaller than the full size L1 (Fig. 2B left panel). All three cell lines exhibited the approximate 32 kDa transmembrane and cytoplasmic fragment resulting from L1 ectodomain shedding by ADAM proteolysis. However, the additional lower weight bands in extracts from 9L/LacZ and U-87/LacZ cells indicates that further, but distinct, proteolysis occurred in these cells; additional lower weight bands were not always consistent (see Fig. 5B for 9L/LacZ). This suggests additional proteolysis of the L1 ectodomain, possibly by plasmin [57]. Surprisingly, antibody UJ127 detected a band approximately the size of full length L1 in U-87/LacZ cell line (Fig. 2B right panel). Since the NCAM-L1 antibody did not detect full length membrane-bound L1 (200-220 kDa) in U-87/LacZ cells, the high molecular weight band detected using UJ127 must be the full size ectodomain (approx. 190 kDa) after ADAM10 cleavage, which runs at approximately the same size on our gels. Taken as a whole, significant proteolysis of L1 occurred in our three glioma cell lines that was undetectable in the surgical samples.

Cell lines C6, 9L/LacZ and U-87/LacZ were immunofluorescently stained on coverslips for L1 using a polyclonal antibody, which targets the L1 ectodomain (Fig. 2C; Lagenaur; [58]). This antibody did not detect surface L1 in C6, 9L/LacZ or U87/LacZ, however a strong punctate cytoplasmic staining of L1 was observed in fixed and permeablized cells (top panels). No cell surface staining was evident even when using a 60× oil immersion objective lens (N.A. = 1.4). As a positive control, this antibody did detect robust cell surface L1 in live mouse L1-transfected quail QT6 cells even at low power (Fig. 2C, rightmost top panel), whereas no staining occurred in untransfected QT6 cells (rightmost bottom panel). We also performed immunostaining after growing cells in the protease inhibitor GM6001 at up to 100 μM, which is 10× the concentration that inhibited cleavage of L1 by other workers in other cancer cell lines. This did not result in the appearance of surface staining (data not shown), which suggests that L1 in these glioma cells is proteolyzed intracellularly where the protease inhibitor does not have access.

Antibody NCAM-L1, which was raised against a C-terminal region peptide sequence of L1, also detected only the cytoplasmic L1 in fixed/permeablized U-87/LacZ cells (Fig. 2C, lower left panel). These results indicate that proteolysis events of L1 occur in glioma cells that are consistent with proteolysis by ADAM family members and plasmin. Interestingly, in U-87/LacZ cells after live staining using the Lagenaur antibody, a punctate staining was observed on the level of the coverslips (Fig. 2C, lower center panel). This is interpreted to be L1 containing exosomal vesicles that are released from these cells (see below), which would be observable as bright immunopositive puncta, although we did not co-stain these for an exosomal marker.

FACS analysis using the Lagenauer antibody also demonstrated L1 expression as distinct peaks in fixed and permeablized C6/LacZ and U-87/LacZ cells (Fig. 2D, left graphs). However, little, if any, surface L1 was observed in live cells (Fig. 2D, right graphs), as no primary negative control cells and L1-stained cells exhibited the same or nearly the same staining patterns. This analysis corroborates immunostaining of cells on coverslips above to demonstrate L1 proteolysis events in C6/LacZ and U-87/LacZ cells to the extent that little or no L1 resides on the surface of these cells. Overall, our data collectively show that L1 expression occurs in the glioma cell lines examined and that L1 is extensively proteolyzed in those cells as well.

### L1 is a component of exosomes

**Figure 3:**
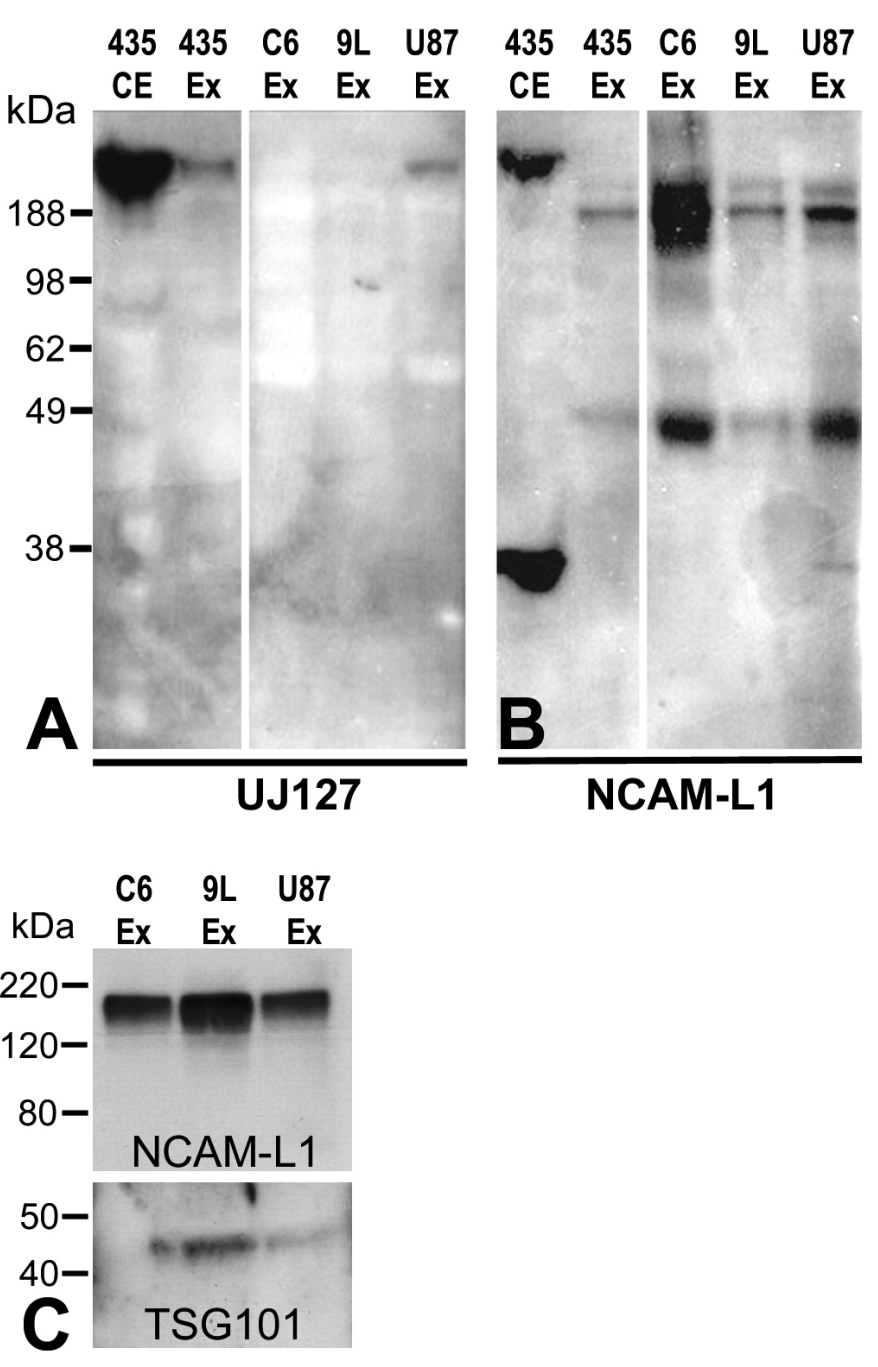
**L1 is present in exosomes**. Rat and human glioma cells were cultured in serum-free media overnight and the media was put through 3 rounds of centrifugation as described to isolate exosomes. The resulting pellets were resuspended and analyzed by western blot with anti-L1 antibodies UJ127 and NCAM-L1. Full-length L1 was detected in exosomes released only from U87/LacZ human glioma cell line using UJ127 (A), whereas smaller weight proteolyzed forms were detected in exosomes from all 3 cell lines using NCAM-L1 (B). The 32 kDa transmembrane fragment was detected only in U-87/LacZ exosomes (B). A cell extract (435 CE) and exosome preparation (435 Ex) from human breast cancer cell line MDA-MB-435 served as a positive control for L1 detection in cells and exosomes. Exosome preps were analyzed for exosomal marker Tsg101 as well as for L1 in (C). Each exosome preparation showed the predicted 45 kDa band for TSG101.

An established exosome isolation protocol [59] was used to isolate exosomes from C6/LacZ, 9L/LacZ, and U-87/LacZ glioma cell line culture media. At the end of multiple rounds of centrifugation, the exosome pellet was resuspended in PBS/PI and analyzed by western blotting for the presence of L1. L1 was detected in exosomes from all 3 glioma cell lines, although the patterns were different from corresponding cell extracts. Antibody UJ127, which is specific for human L1, detected full length L1 in exosomes from U-87/LacZ cells (Fig. 3A) but expectedly not from the two rat cell lines. In all three cell lines, the presence of L1 was found in exosomes using the NCAM-L1 antibody (Fig. 3B). However the small membrane/cytoplasmic fragment remaining after ectodomain shedding was found only in U87/LacZ cell exosome preparation. A cell extract and exosome preparation from human breast cancer cell line MDA-MB-435 served as a positive control for L1 detection in cells and exosomes (based on unpublished data; manuscript in preparation). The cell extract resulted in robust full length and cytoplasmic fragment staining, and the exosome preparation resulted in staining similar to that from U-87/LacZ cells using the UJ127 antibody and to that from C6/LacZ and 9L/LacZ cells using the NCAM-L1 antibody. Exosome preparations were also evaluated for the exosomal marker protein TSG101, and each showed the presence of this marker (Fig. 3C). This analysis showed that L1 can be released from glioma cells as a component of exosomes, which suggests that exosomes might serve as another source of soluble L1 in extracellular space to interact with glioma cell surface integrins.

### L1-binding integrins are expressed in glioma cell lines

**Figure 4:**
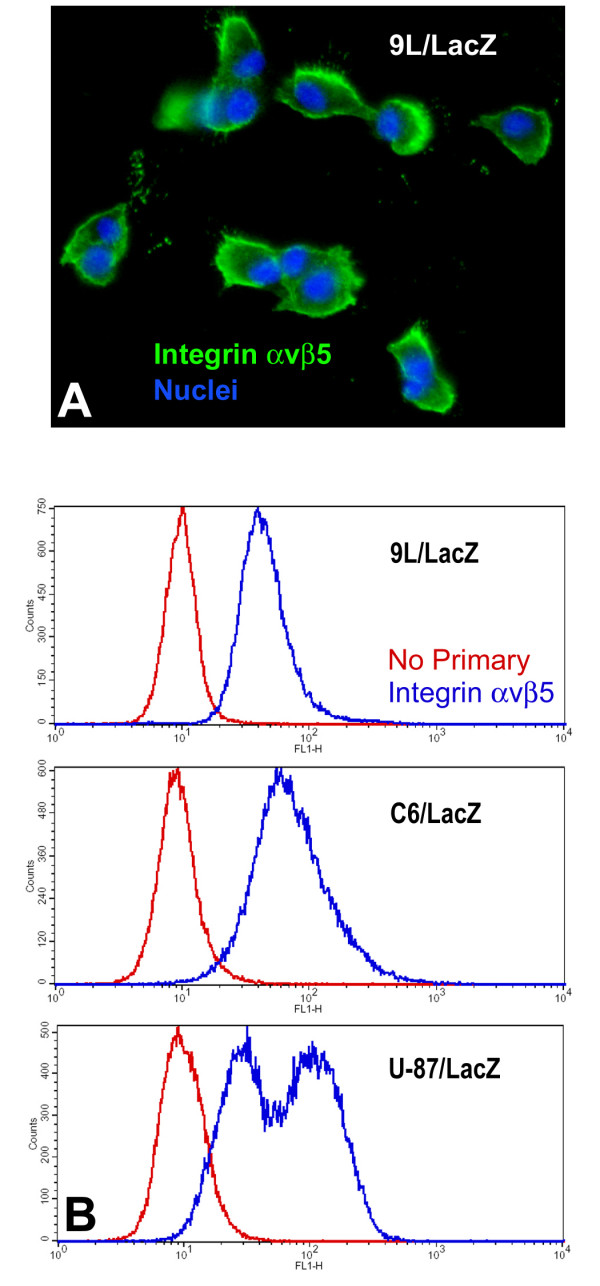
**L1-binding integrin expression**. (A) 9L/LacZ cells were immunostained live with a mouse monoclonal antibody against integrin αvβ5 (green) and nuclei were counterstained with bisbenzimide (blue). Cell surface staining for integrin αvβ5 is clearly visible. (B) Rat glioma cells 9L/LacZ and C6/LacZ and human glioma cells U87/LacZ were immunostained live for integrin αvβ5 and analyzed by flow cytometry. All 3 cell lines exhibited distinct positive peaks for integrin αvβ5 (blue lines) compared to no primary antibody negative controls (red lines), and U87/LacZ cells appeared to have two overlapping positive populations: one high-level positive population and another lower-lever positive population.

The RGD motif within the 6^th ^Ig domain of L1 ectodomain can be recognized by several RGD-binding integrins on the cell surface. Here, one of the L1-binding integrins, αvβ5, was examined in the three glioma cell lines. 9L/LacZ cells were immunofluorescently stained live on coverslips to visualize cell surface staining for αvβ5 (Fig. 4A). These cells exhibited clear cell surface staining, which was most prominent at cell edges. 9L/LacZ, C6/LacZ, and U87/LacZ were also immunostained live in suspension for quantitation of αvβ5 by FACS analysis (Fig. 4B). All three cell lines exhibited distinct positive peaks for integrin αvβ5 (blue lines) compared to no primary antibody negative controls (red lines), and U87/LacZ cells appeared to have two different positive populations. This would represent one high-level positive population and another low-level positive population. Thus, this evidence of the L1-binding integrin present in these gliomas cell lines, along with L1 ectodomain proteolysis, indicates that the RGD-containing ectodomain of L1 could stimulate glioma cell motility by triggering cell signaling pathways by interacting with L1-binding integrins.

### Antisense-L1 retroviral vector decreased cell motility in 9L/lacZ cells

**Figure 5:**
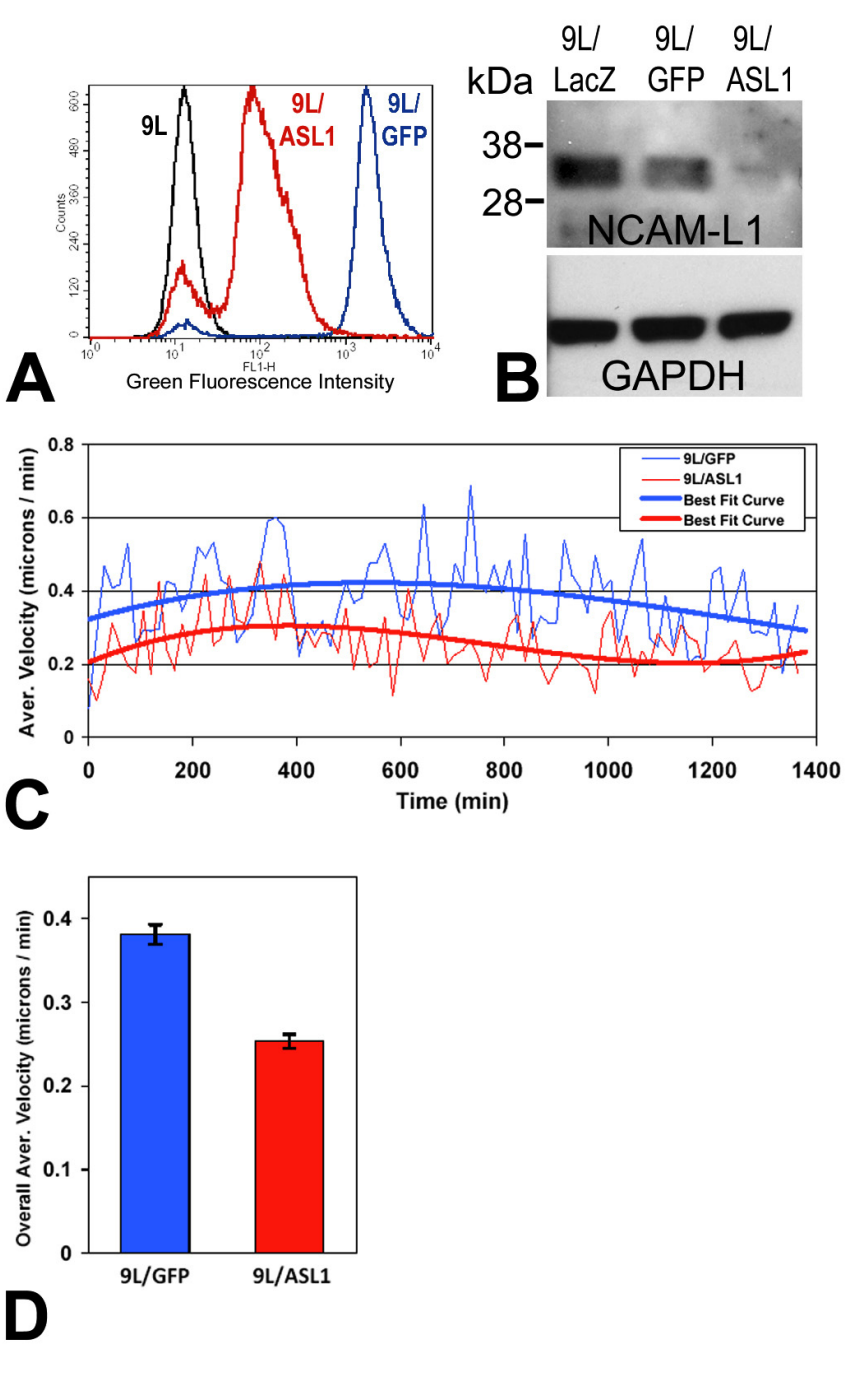
**L1-attenuated glioma cells were less motile**. (A) 9L/LacZ cells were infected with mouse antisense-L1 retroviral vector or control pLEGFP-C1 vector. The positively infected cells were then sorted by FACS. Post-sort analysis of live cells is shown with fluorescence intensity level displayed along the X-axis. The black peak represents uninfected cells, the blue line represents pLEGFP-C1 vector infected negative control cells (96% infected), and the red line represent the cells infected with antisense-L1 vector (87% infected). Antisense-L1 infected cells were less bright green than the extremely bright control GFP-only cells, presumably because of fusion of GFP to the antisense sequences. (B) L1 expression was analyzed by western blot in vector infected 9L cells purified above. Whole cell lysates were made and the same amount of total protein was loaded into each lane (30 μg). Western blots were stained with NCAM-L1 polyclonal anti-cytoplasmic L1 and the density of the 32 kDa cytoplasmic L1 fragment was analyzed using Un-Scan-It software. The 32 kDa cytoplasmic fragment was reduced by approximately 90% in the antisense infected 9L/ASL1 cells compared to the 9L/GFP negative control cells. Blots were probed for GAPDH, which was used to normalize protein levels for quantitation of L1. (C) Cells were assessed by the *Super Scratch *assay for effects on cell motility. Antisense-L1 infected 9L/lacZ cells showed decreased motility (red graph) compared to pLEGFP infected control cells (blue graph). The time-lapse graph shows the average velocities (thin lines) and 3^rd ^order polynomial best-fit curves (thick lines) of control and antisense-L1 infected populations of cells analyzed above at 15 min. intervals. The average velocity of the antisense-infected cells was consistently lower throughout the time course of the experiment. (D) The overall average velocities were calculated using all individual cell velocities collected during the course of the experiment and graphed as single values for the two populations of cells. Control GFP-only infected cell velocity was 0.381 ± 0.012 s.e.m. and AS-L1 infected cell velocity was 0.253 ± 0.008 s.e.m. The differences in the velocities are highly significant (p < 0.001). The experiment was repeated twice with similar results.

An antisense-L1 retroviral vector was constructed to target the first 530 bp of L1 including the L1 translation start codon in order to attenuate L1 expression in infected cells, similarly to our studies using retroviral antisense sequences targeted to integrin subunits [60,61]. The antisense sequence is located after the EGFP sequence, which generates an antisense mRNA "tail" after EGFP. This allows the antisense mRNA to attenuate L1 translation and at the same time allows EGFP expression. 9L/LacZ rat glioma cells were infected with either the antisense-L1-GFP vector or control GFP vector. Because infections of cell cultures were incomplete (approximately 50%), the infected cells were sorted by FACS for GFP fluorescence to increase percentages of GFP-positive infected cells. FACS sorting resulted in 96% GFP vector infected cells (9L/GFP cells) and 87% antisense-L1-GFP infected cells (9L/ASL1) based on GFP fluorescence (Fig. 5A). Western blotting was used to evaluate attenuation of L1 expression in the antisense vector infected and sorted 9L/LacZ cells. The NCAM-L1 antibody was used to detect the 32 kDa L1 transmembrane/cytoplasmic domain and quantitation was performed. Compared to GFP vector infected cells (9L/GFP), the antisense-L1-GFP infected cells (9L/ASL1) were reduced in L1 levels by approximately 90% (Fig. 5B).

After confirming that L1 expression was significantly decreased in L1 antisense vector-infected cells, quantitative motility analyses were performed using time-lapse microscopy of cells along the edges of scratches made in confluent monolayer cultures (*Super Scratch *Assay; [62]). Cells infected with the antisense-L1-GFP vector migrated into the "wound" at a lower average velocity throughout the experiment (Fig. 5C) and significantly reduced overall average velocity (0.381 μm/min.) compared to cells infected with the GFP vector (0.253 μm/min.; 34% reduction; p < 0.001) (Fig. 5D). Overall average velocity measurements are the averages of the velocities of 20 cells at 15 minute intervals over 23 hours, resulting in the average of approximately 1800 velocity values for each condition. These results show that the antisense L1 vector, which decreased L1 expression in 9L/LacZ cells, significantly decreased cell motility rates as well. The slower motility rate of L1-attenuated cells suggests that it was the released soluble L1 ectodomain and/or released L1 containing exosomes that stimulated their motility above their basal level in an autocrine fashion.

### Anti-L1 antibodies decreased cell motility

**Figure 6:**
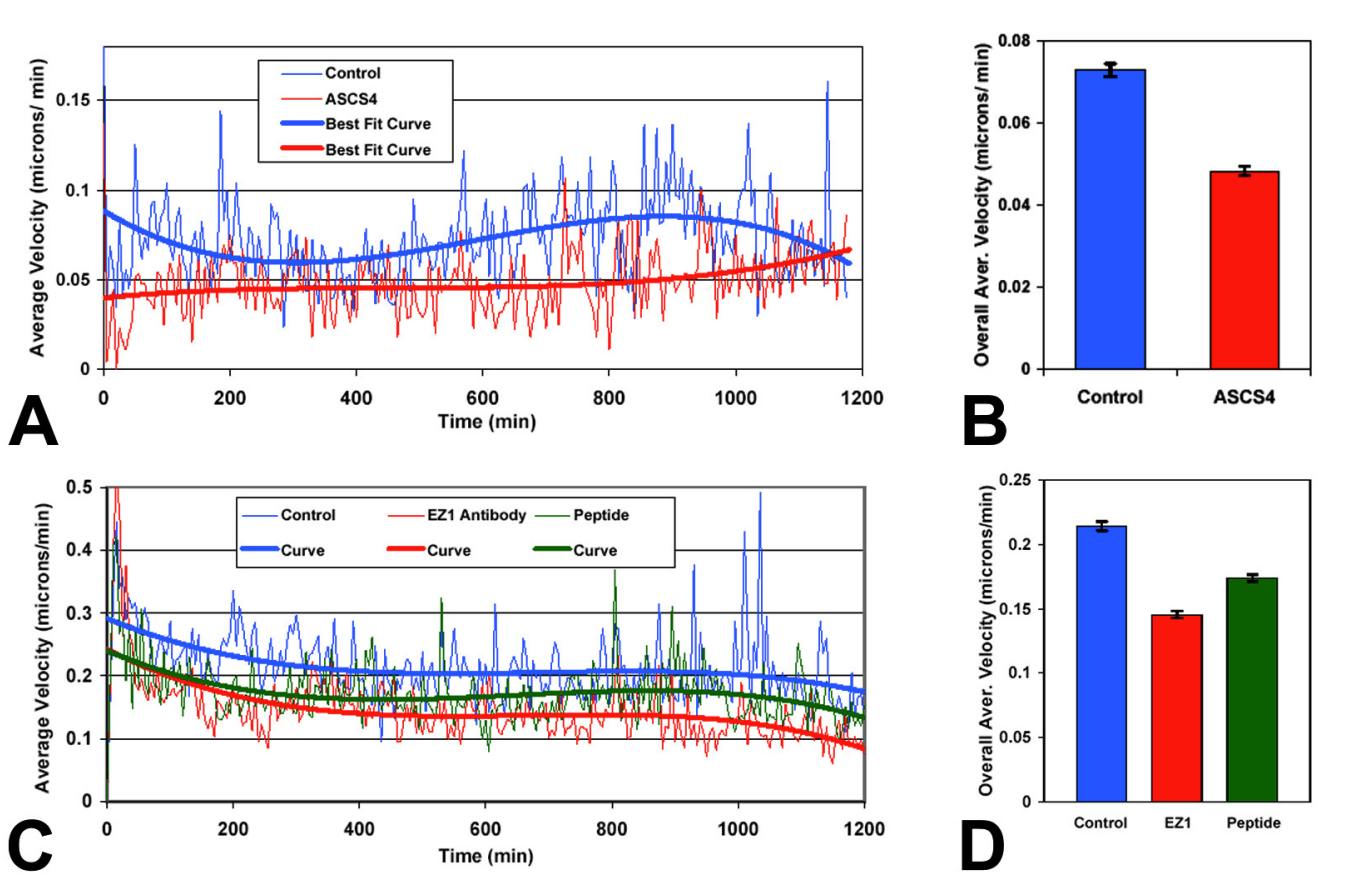
**Anti-L1 antibodies and RGD peptide inhibit glioma cell motility**. (A) Anti-L1 antibody ASCS4 was added to 9L/LacZ cultures and assessed by the *Super Scratch *assay for effects on cell motility. The time-lapse graph shows the average velocities and best-fit curves of the control and antibody treated 9L/Lacz cells over a 20 hour time period (15 cells each condition). The antibody ASCS4 consistently lowered the velocities of migrating cells throughout the course of the experiment. (B) The overall average velocities were calculated using all individual cell velocities collected during the course of the experiment and graphed as single values for the two populations of cells. Control cell velocity was 0.073 ± 0.002 μm/min. Velocity of the ASCS4 treated cells was reduced 34% compared to untreated controls at 0.048 ± 0.001 μm/min (p < 0.001). Bars are s.e.m. (C) Anti-L1 antibody EZ1 or the RGD-containing peptide antigen were added to cell cultures as described and assessed by the *Super Scratch *assay for effects on 9L/LacZ cell motility. The time lapse graph shows the average velocities and best-fit curves of the control, antibody treated, and peptide treated 9L/Lacz cells over a 20 hour time period (2 areas of interest and 40 cells each condition). The antibody EZ1 and peptide consistently lowered the velocities of migrating cells throughout the course of the experiment, with the antibody having a greater effect on reducing migration velocity. (D) The overall average velocity for the three populations of cells from (C) is shown. Control cell velocity was 0.214 ± 0.003 μm/min. Velocity of the EZ1 treated cells was reduced 32% (0.146 ± 0.003 μm/min) and velocity of peptide treated cells was reduced by 19% (0.174 ± 0.003 μm/min) compared to untreated controls (p < 0.001 for each comparison). Bars are s.e.m. Shown are data from one representative experiment. The experiments were repeated twice with similar results.

As an alternative method to inhibit L1 autocrine stimulation of motility, antibodies specific for the rat L1 ectodomain (ASCS4; [63,64]) and an antibody specifically targeting the L1 RGD motif in the 6^th ^Ig domain (EZ1; see Methods) were used with 9L/LacZ rat glioma cells in the *Super Scratch *assay. Quantitation of cell motility was determined after time-lapse microscopy as described. The average cell velocities over time of treated vs. untreated cells and the 3^rd ^order polynomial curves of best fit were plotted for the course of the experiment (Fig. 6A). The velocity of 9L/LacZ cells in the culture containing ASCS4 was consistently reduced throughout the time course of the experiment. The overall average velocity of 9L/LacZ cells incubated with ASCS4 was decreased substantially and significantly compared to the untreated control cells (34%; p < 0.001; Fig. 6B), and similarly to cells with attenuated L1 above. Overall average velocity measurements were the averages of the velocities of approximately 20 cells at 5 minute intervals over approximately 20 hours, resulting in the average of approximately 4800 velocity values for each condition. The overall average velocity of 9L/Lacz cells incubated with EZ1 also was decreased similarly and consistently (Fig. 6C, red), which resulted in an overall decrease in motility of 32% (Fig. 6D; p < 0.001). Overall average velocity measurements were the averages of the velocities of approximately 40 cells at 5 minute intervals over approximately 20 hours, resulting in the average of approximately 9600 velocity values for each condition. In contrast, we have performed time-lapse motility experiments using the Lagenaur polyclonal antibody, and no decrease in motility was observed (data not shown), which suggests that it binds to the L1 ectodomain at a non-critical domain. Since 9L/LacZ cells do not express surface L1 (Fig. 2; and [65]), ASCS4 and EZ1 acted most likely through blocking the soluble L1 ectodomain and/or exosomal vesicle interaction with integrin receptors and, thus, interfering with autocrine stimulation of motility.

The L1 peptide used to generate EZ1, containing the RGD motif of L1, was also used to treat cells to antagonize the binding of soluble L1 with integrins on the cell surface in the *Super Scratch *assay. The peptide also decreased the motility of 9L/LacZ cells consistently, but less than the EZ1 antibody (Fig 6C, green). The overall average cell velocity was decreased by 19% (Fig. 6D, green; p < 0.001). Overall average velocity measurements were the averages of the velocities of approximately 40 cells at 5 minute intervals over approximately 20 hours, resulting in the average of approximately 9600 velocity values. The ability of the soluble L1 peptide to significantly inhibit motility was not surprising and provides further evidence that L1 stimulates glioma cell motility, primarily through the soluble ectodomain and/or L1-containing exosomes.

## Discussion

In our study, L1 (L1CAM; CD171) expression was documented in high-grade human glioma surgical samples and proteolyzed L1 ectodomain was released into the media when these cells were cultured. Glioma cell lines from two different species (rat and human) also expressed L1, and proteolysis occurred most likely by ADAM10 to release a large ectodomain. L1-binding integrins also were detected in those cells, which raised the possibility that an autocrine stimulatory mechanism could be operating. Glioma cell motility was reduced by using antisense retroviral vector attenuation of L1 expression and blocking antibodies against the L1 ectodomain. These experiments provide evidence that in these glioma cells L1 is cleaved and released to stimulate cell motility through an autocrine/paracrine mechanism.

Malignant glioma is the most common human brain cancer [1]. The most aggressive type of glioma is glioblastoma multiforme (GBM), which can kill patients within months [66]. Although metastasis outside the CNS is rare among GBM, the cancer is extremely invasive and can migrate rapidly inside the brain. Previous studies by others showed L1 expression in some glioma cell lines and primary glioma, and it has been suggested that L1 facilitates glioma cell migration within the brain [4,46-48,67]. However, L1 proteolysis needed to be characterized and quantitative experiments needed to be performed to test the hypothesis that L1 facilitated glioma cell motility by an autocrine/paracrine mechanism.

Our study confirmed and considerably extended previous studies on expression and proteolysis of neural adhesion molecule L1 in high-grade human glioma surgical samples and in rat and human glioma cell lines by using RT-PCR, western blotting, immunofluorescent staining, and FACS analysis. L1 was detected in 17 out of 18 surgical samples analyzed. This suggests that L1 may be a marker for high-grade gliomas and that it may play a significant functional role in the behavior of these cells. However, by western blot analyses of primary surgical specimens we could not detect that L1 was proteolyzed to generate an autocrine stimulatory ectodomain, in contrast to the proteolysis that occurred in cell lines. This might be because our samples were from the interior of the tumor, not the margin, where the cells are densely packed and no free edge exists where the cells are invading the brain. L1 and its protease ADAM10 have been shown in colon cancer only at the invasive front [31], where proteolysis occurs to stimulate cell migration. We did detect L1 ectodomain in culture supernatant from surgical specimen cells. It is possible that gliomas are similarly autocrine stimulated by proteolyzed L1 ectodomain only at the tumor margin or when cells are not in a dense tumor mass such as in culture. However, control mechanisms may be different since active ADAM10 was expressed in all of our surgical samples, albeit only at a high level in one sample (# 10). Either the levels of active ADAM10 were not sufficient in the surgical samples to generate detectable L1 ectodomain, or L1 proteolysis was inhibited, possibly by its inclusion in intercellular junctions [68] in the interior of the tumor mass. At this time, the lack of L1 ectodomain in the surgical samples along with active ADAM10 requires further investigation.

L1 could only be visualized intracellularly in fixed glioma cell lines, with surface staining lacking in both live and fixed cells by immunocytochemistry and FACS analysis. These results suggested the cleavage of surface L1 by its known surface protease, ADAM10 [35], to release the soluble L1 ectodomain. The cell surface L1-binding integrin αvβ5 was found on the three glioma cell lines used here. This integrin previously was shown to interact with L1 and to be involved in L1-enhanced cell migration [33]. Integrin αvβ5 detection in the cell lines indicates the possibility that soluble L1 ectodomain could interact with this integrin receptor.

L1 ectodomain stimulation in glioma cells was demonstrated by using a highly quantitative time-lapse motility assay [62,65], which allowed tracking of individual cell migration distances and velocities throughout the experiment. Motility decreased significantly when an antisense retroviral vector was used to attenuate overall L1 expression in 9L/LacZ cells. We have used this antisense strategy previously with success to attenuate integrin subunits in normal neuroblasts in developing brain and to implicate them in neuronal migration [60,61]. Since there is no surface L1 on these cells, this strategy along with the antibody blocking experiments to sequester the soluble L1 ectodomain, provides evidence that an autocrine stimulation of motility occurs in these glioma cell lines. Our collective results demonstrated that the soluble L1 ectodomain, and not surface L1, stimulated cell motility in glioma cell lines. This suggests that autocrine/paracrine stimulation by the soluble L1 ectodomain might be a mechanism that operates during pathologic diffuse glioma cell migration, for which a mechanism has still not been elucidated.

Our findings are different from those of Senner et al. (2002) [69] where they found no intrinsic L1 expression in their C6 rat glioma cell line. In our studies, the C6 cell line and its two derivatives (C6/lacZ and C6/LacZ7) not only expressed L1, but also proteolyzed it, presumably by ADAM10. We suppose that either their cultures of C6 cells lost L1 expression as a whole, the cell clone they picked was a non-expressing variant of L1 expression, or that their inability to detect L1 was a result of their antibody choice. We have found that the several different anti-L1 antibodies we used are individually suited for particular assays, and none are best for all. Thus, their findings that ectopic expression of L1 in C6 cells resulted in no significant difference in migration on either matrigel or myelin compared to plain C6 cells might be because the cells already expressed L1, but they did not detect it. It is equally possible that our particular cultures of C6 and derivatives actually do differ in L1 expression from their particular cultures, for unknown reasons. Nonetheless, our C6 cell lines were found to express intrinsic L1 and to cleave it.

Previously, we found that C6 cell motility was significantly decreased when those cells were plated on cell monolayers that ectopically expressed a non-cleaved cell surface NgCAM/L1 (3T3/NgCAM cells; [62]). Thus, their autocrine stimulation by intrinsic L1 ectodomain appeared to be overridden by heterophilic adhesive interactions between the extrinsic NgCAM on the 3T3 cell surfaces, presumably with integrin receptors. In addition, ectopic expression of cell surface NgCAM/L1 directly in 9L/lacZ cells, which we have shown here also express and cleave intrinsic L1, decreased their motility significantly [65], which suggests that surface L1 is not stimulatory even on a background of cleaved L1. In all, these experiments suggest that L1 is stimulatory for motility only when predominantly cleaved to form a soluble ectodomain. However, when also present in the uncleaved cell surface form it reduces motility due to adhesive interactions that override any stimulatory effect of the ectodomain. Thus, we propose a model whereby for high-grade glioma cells to migrate away from the tumor mass and to be invasive, they must proteolyze L1 to cause autocrine stimulation. Otherwise, the cells would be strongly adherent via their cell surface L1-L1 and L1-integrin interactions.

## Conclusion

Our novel results support a model of autocrine/paracrine stimulation of cell motility in glioma cells by a cleaved L1 ectodomain and/or released exosomal vesicles containing L1. This mechanism might explain the diffuse migratory behavior of high-grade glioma cancer cells within the brain, where they apparently do not follow discernable anatomical structures. We found L1 in a high percentage of high-grade glioma surgical specimens and shed L1 ectodomain in their cell culture, which suggests a potential function in these cells. Established glioma cell lines expressed and proteolyzed L1 to release a soluble ectodomain and also released minute exosomal vesicles that contained L1, both of which could serve as diffusible sources of L1 that stimulate the migration of these cells in an autocrine/paracrine manner. Cell lines also expressed an L1-binding integrin receptor, which could mediate necessary intracellular signaling events. Functional studies confirmed that L1 expression and ectodomain release was responsible for increased glioma cell motility.

## Methods

### Primary glioma surgical samples and cell lines

A total of 18 high-grade (Grade III and GBM) human primary glioma surgical samples were obtained through the Tissue Procurement Center at the Helen F. Graham Cancer Center, Christiana Care (Newark, DE) following approved protocols and analyzed by western blotting for L1 expression. The surgical samples were transported in Hibernate A medium (BrainBits LLC, Springfield, IL) on ice. The samples were washed in ice-cold neurobasal medium (Invitrogen, Carlsbad, CA), the visible necrotic sites of the tumor were trimmed, and the remaining tissue was cut into <1 mm^3 ^pieces. Pieces from 14 surgical samples were lysed directly in RIPA lysis buffer (150 mM NaCl, 1% TX-100, 1% SDS, 0.5% sodium deoxycholate, 50 mM Tris pH 7.5) with Complete Mini EDTA-Free Protease Inhibitor cocktail tablets (PI) (Roche Diagnostics, Indianapolis, IN) followed by sonication for western blot analysis. Pieces from 4 surgical samples were rinsed in neurobasal medium and then incubated at 37°C in 5 ml of 0.25% trypsin-EDTA (Mediatech Inc., Herndon, VA) before sample dissociation into single cells. The enzymatic digestion was terminated by 5 ml of 0.03% SBTI (Soybean Trypsin Inhibitor) and 0.003% DNase I on ice for 10 minutes before the sample was centrifuged. The supernatant was removed after centrifugation and 2 ml of SBTI/DNase was added to resuspend the pellet. Single cells were dissociated by trituration with a Pasteur pipet. The cells were collected in the supernatant after waiting for 5 minutes on ice to let the non-dissociated tissue pieces sink to the bottom of the tube. Dissociated cells were pelleted by centrifugation and then were cultured in DMEM (Mediatech, Inc., Herndon, VA) with added 10% fetal bovine serum (FBS; Hyclone, Waltham, MA), 2 mM L-glutamine (Mediatech Inc.), and penicillin-streptomycin (Mediatech Inc.). Cell cultures were maintained for 2, 4, and 15 weeks before trypsinization from the culture plates and preparation of samples for western blot analysis for L1 as above. The cells were growing in the culture medium with PI overnight before the medium was collected and filtered through 0.2 μm filter. Then, the filtered cell culture supernatant was further concentrated using a YM-100 protein concentrator (Millipore, Billerica, MA). Some pieces of glioma surgical samples were fixed in 4% paraformaldehyde (Electron Microscopy Sciences, Hatfield, PA) in PBS at room temperature after trimming. The fixed samples were cryoprotected in 30% sucrose in PBS overnight at 4°C and were frozen in tissue freezing medium (Triangle Biomedical Sciences Inc, Durham, NC) in an ultralow freezer (-75°C). The tissue sections were cut at 10 μm thickness on a Leica cryostat and used for immunofluorescent staining below.

Glioma cell lines used in this study were obtained from the American Type Culture Collection (ATCC, Manassas, VA). 9L/LacZ is a rat gliosarcoma cell line [70]. C6/LacZ, C6/LacZ7 [71], and C6 are rat glioma cell lines [72]. U-87/LacZ is a human GBM cell line obtained and described previously [65]. QT6 is a quail fibrosarcoma cell line [73]. CHO is a Chinese hamster ovary cell line [74]. QT6 cells were cultured in Medium 199 (Mediatech, Inc.) with 5% FBS, 20% tryptose phosphate broth, 2 mM L-glutamine and penicillin-streptomycin. Other cell lines were cultured in DMEM (Mediatech, Inc.), 10% BGS (bovine growth serum; Hyclone, Waltham, MA), 2 mM L-glutamine, and penicillin-streptomycin and incubated at 37°C with 5% CO_2_.

### Vectors, transfection and infection

QT6 cells were transfected with a full length mouse L1 expression plasmid with an RSV promoter, LacZ gene and IRES-L1 (1481dL1Nco) or human L1 expression plasmid using standard calcium phosphate transfection method [75]. Transfected cells served as a positive control for L1 expression in western blotting. CHO cells were infected with a lentiviral vector (1879hL1ecto) containing a CMV promoter followed by the complete human L1 ectodomain sequence and served as positive control for detecting human L1 ectodomain.

The mouse L1 antisense retroviral vector was generated with a pLEGFP-C1 expression vector (Clontech, Moutain View, CA). pLEGFP-C1 facilitates retroviral delivery and expression of the enhanced green fluorescent protein (EGFP) or carboxy-terminal fusions of EGFP to a protein of interest. The retroviral elements in pLEGFP-C1 are derived from a Moloney murine leukemia virus (MoMuLV). The antisense sequence is targeting the first 530 bp of mouse L1 cDNA [76] including the start codon of L1 protein translation. The PCR product was generated using the forward primer: 5'-CGG CGG GTC GAC CCT TTG AAA AAC ACG ATG ATA AGC TTG CCA CAA-3' and the reverse primer: 5'CGG CGG AGA TCT GTT TGA TGT CGA AAA TCT TGC TGT TCA TCC AGT-3'. The double CGG are the overhangs designed for easier cloning. GTC GAC is SalI restriction site, AGA TCT is BglII restriction site. Both restriction enzyme sites were used for ligating the antisense L1 sequence into pLEGFP-C1 multiple cloning sites. The antisense sequence is located immediately following the EGFP stop codon, which generated a mouse L1 antisense mRNA "tail" after EGFP transcription. In this way, the "tail" can serve as antisense mRNA to attenuate L1 protein expression, and at the same time it will not interfere with EGFP expression. The vector (10 μg) was then transfected into HEK 293T/17 cell line with the helper plasmid pPAM (10 μg) using standard calcium phosphate method; the viruses were collected 48 and 72 hours after transfection. 9L/LacZ cells were infected with the viruses. The pLEGFP-C1 vector was also transfected into HEK 293T/17 cell line to produce viruses. The 9L/LacZ cells infected with empty pLEGFP-C1 viruses were served as negative control. The positively infected 9L/LacZ cells were sorted on a FACSCalibur flow cytometer (Becton Dickinson, San Jose, CA) for positive GFP fluorescence.

### Antibodies for western blotting, immunofluorescent staining, time-lapse and FACS analysis

The antibodies used here are as follows. NCAM-L1 C-20 (cat. # sc-1508; Santa Cruz Biotechnology, Santa Cruz, CA) is a goat polyclonal antibody raised against a peptide mapping within a C-terminal cytoplasmic domain of L1CAM of human origin. UJ127 (cat. # GTX72362; Gene Tex, Irvine, CA) is a mouse monoclonal antibody raised against the human ectodomain of L1 (within the fibronectin repeats and close to the membrane region). Lagenaur anti-L1 is a rabbit polyclonal antibody raised against the whole L1 molecule and reacts with the ectodomain of mouse, rat and human L1 [77], and was generously provided by Dr. Carl Lagenaur (Univ. of Pittsburgh). ASCS4 (Developmental Studies Hybridoma Bank, DSHB) is a mouse monoclonal anti-rat L1 ectodomain antibody [63,64]. It does not react with human L1. EZ1 is a custom rabbit polyclonal antibody generated by targeting a 15 amino acid human L1 peptide sequence provided to EZBiolab (Westfield, IN). The peptide sequence "QPSITWRGDGRDLQEC" was centered on the integrin binding RGD sequence in the 6th Ig domain (extracellular region). This was done for the purpose of raising function blocking antibodies that compete with L1 ectodomain to interact with RGD-binding integrins in cell migration assay. This sequence was found only in L1 when analyzed by BLAST (National Institutes of Health).

Anti-ADAM10 (cat. # 28071; AnaSpec, Inc., Fremont, CA) was a rabbit polyclonal antibody. Mouse anti-human integrin αVβ5 (cat. # MAB2019Z; Chemicon International, Billerica, MA) was a mouse monoclonal antibody which reacts with the human integrin αVβ5 and binds to the ligand binding site. Several different loading controls (GAPDH, β-tubulin, α-actin, and HSP70) were used. None of these gave results consistent with the BCA assay for surgical glioma specimens, and all resulted in different strength bands relative to each other. This discrepancy was not seen with the glioma cell lines, and we suspect that none of these loading controls is more accurate than the BCA assay for surgical gliomas, probably due to each loading control marker being expressed variably in the different surgical gliomas. The GAPDH antibody (cat. # 2118; Cell Signaling, Boston, MA) is presented as the loading control in this paper. The monoclonal mouse anti-TSG101 antibody (4A10; cat. # ab83; Abcam, Cambridge, MA) was used as the exosome marker. To detect GFAP, a polyclonal rabbit anti-GFAP antibody was used (cat. # 18-0063; ZYMED Laboratories, South San Francisco, CA). Anti-NF-M (cat. # IMG-5076A-2; IMGENEX, San Diego, CA), a polyclonal rabbit anti-neurofilament (recombinant) antibody, was used to detect medium neurofilament in the surgical samples. To detect mitotic cells, a rabbit polyclonal anti-Ki67 antibody (cat. # ab15580; Abcam, Cambridge, MA) was used followed by Alexa Fluor-594 secondary antibody. Secondary antibodies used for L1 immunostaining were HRP-conjugated donkey anti-goat IgG (Jackson Immunoresearch, West Grove, PA), Alexa Fluor-488 or -594 donkey anti goat IgG (Molecular Probes, Invitrogen, Carlsbad, CA), HRP-conjugated goat anti-mouse IgG (Jackson Immunoresearch), or Alexa Fluor- 488 or -594 goat anti mouse IgG (Molecular Probes, Invitrogen). For integrin receptor and live cell L1 immunostaining a biotin-conjugated goat anti-mouse or anti-rabbit IgG (Jackson Immunoresearch) followed by Alexa Fluor-488 streptavidin conjugate (Molecular Probes, Invitrogen) were used.

### RT-PCR

The total RNA was extracted from the cell lines using the RNeasy Mini Kit (Qiagen, Valencia, CA). The first-strand cDNA synthesis was carried out using the SuperScript III First-Strand Synthesis System for RT-PCR (Invitrogen). The primer sequences used were designed against the transmembrane domain of L1 conserved in both humans and mice. The forward primer sequence was 5'-TACCGCTTCCAGCTTCAG and the reverse sequence was 5'-TGATGAAGCAGAGGATGAGC. PCR Master Mix (Invitrogen) was used and the reaction was carried out in a thermal cycler (Techne, Burlington, NJ) using an initial denaturation step for 15 min at 95°C, after which the reactions were subjected to 40 cycles of amplification. Each cycle consisted of denaturation for 30 seconds at 94°C, annealing for 30 seconds at 55°C and extension for 1 min at 72°C. The final extension was for 10 min at 72°C and the final hold was at 4°C.

### Western blotting

To make protein extracts, cells culture dishes were kept cold on ice for 10 min and rinsed with cold PBS/PI (Phosphate buffered saline with protease inhibitors) before solublizing them in RIPA lysis buffer with PI for 2-3 min on ice. The cells were then scraped, collected in microcentrifuge tubes and the lysates were clarified by sonication at 10% power using a Sonic Dismembrator (Model 500, Fisher Scientific, Pittsburgh, PA). The primary human glioma surgical samples were minced and put into RIPA lysis buffer as explained previously; lysates were sonicated at 10% power as well. Protein quantification was performed using the BCA Assay (Pierce Biotechnology, Pittsburgh, PA). The samples were prepared by adding the NuPage 4× LDS sample buffer (Invitrogen) and the NuPage 10× reducing agent (Invitrogen), heating them at 70°C for 10 min and centrifuging them. Equal amounts of the proteins (8-30 μg, depending on experiment) were loaded on NuPage 4-12% gradient polyacrylamide gels (Invitrogen) along with protein ladders (See Blue Plus 2 prestained standard, Magic Mark XP, Invitrogen). A small volume (500 μl) of the NuPage antioxidant (Invitrogen) was also added to the top chamber of the running buffer tank (OWL Separation Systems, Inc, Waltham, MA) prior to the electrophoresis to prevent the proteins from reoxidizing during electrophoresis. Running buffer used was the NuPage MOPS running buffer (Invitrogen).

For Western transfer, polyvinylidine fluoride (PVDF) membranes (0.45 μm, Invitrogen) were treated in methanol for 30 sec- 1 min and rinsed with water in order to make them hydrophilic. Transfer buffer used was the NuPage 20× transfer buffer (Invitrogen) with 20% methanol and 1.4 ml of the NuPage antioxidant. The transfer was carried out at 4°C at 30 V overnight in a Fisher model 172538 transfer apparatus. The membrane was blocked in the blocking solution (5% nonfat dry milk solution in Phosphate Buffered Saline with 0.01% Tween20) rotating or rocking for either 1-2 hours at room temperature or overnight at 4°C. The blots were then incubated in the primary antibody, either rocking for 1-2 hours at room temperature or overnight at 4°C. HRP-conjugated secondary antibody was then added to the membranes and kept on a rotator or rocker for 1 hour at room temperature. The membranes were developed for 1 to 2 min using the enhanced chemiluminescence detection system (Amersham, Piscataway, NJ or Pierce Biotechnology, Pittsburgh, PA); the protein bands in the blots were finally visualized by exposing the Blue Basic Autorad Film (ISC Bioexpress, Kensville, UT) for various time intervals.

### Immunofluorescent staining

Fixed cell staining was carried out by growing cells on coverslips previously coated with 200 μg/ml poly-L-ornithine (Sigma-Aldrich, St. Louis, MO) and then first fixing them in 1% formaldehyde in PBS for half an hour at room temperature. The coverslips were then rinsed twice in PBS and the cells were incubated in the primary antibody diluted in PBS + 5% FBS or 5% normal goat serum + 0.03% Triton X-100 for an hour at room temperature. The coverslips were then rinsed twice with PBS and incubated in the Alexa Flour 488 or 594 secondary antibodies (Molecular Probes, Invitrogen) in the same diluents as the primary antibodies for 45 min-1 hr at room temperature. The coverslips were again rinsed in PBS and incubated in 10 μg/ml bisbenzimide (Sigma-Aldrich) in PBS for 5-10 min. The cells were rinsed again in PBS and the coverslips were then mounted in 6 μl buffered glycerol/para-phenylenediamine (pH 8.0) on glass slides. For live cell staining for L1 or integrin, instead of fixing the cells initially, cells were incubated in primary antibody in DMEM + 5% heat-inactivated FBS on ice, rinsed, fixed with 1% formaldehyde at room temperature for 30 minutes, rinsed, incubated in biotin-conjugated secondary antibody in PBS + 5% NGS, rinsed, incubated in Alexa 488-streptavidin, and rinsed before the nuclear staining and mounting. The staining was visualized using a Nikon Microphot-FX microscope with Fluor objectives.

### FACS analysis

The cells were trypsinized lightly in 0.05% Trypsin/0.02% EDTA for several minutes, resuspended in DMEM + 10% H-I serum and centrifuged at 1000-1200 × g (700-800 rpm Beckman Allegra 6R centrifuge). The supernatant was aspirated and the cell pellet was resuspended in DMEM + 10% H-I serum. For fixed cell L1 staining, the cells were fixed first with 1% formaldehyde (as described above) followed by Lagenaur anti-L1 primary and Alexa Fluor-488 secondary antibody incubation. For live cell staining for L1 and integrins, cells were incubated with primary in the heat-inactivated serum containing diluents on ice (as described above), fixed, incubated in a biotin-conjugated secondary antibody followed by Alexa Fluor-488 streptavidin conjugate (Molecular Probes, Invitrogen). The cells then were examined by a Becton Dickinson FACSCalibur flow cytometer for immunofluorescence levels using Cell Quest software.

### Exosome isolation

Ultracentrifugation was performed to isolate the exosomes from the culture media of the glioma cell lines. Briefly, the glioma cells were seeded on 10 cm dishes with serum-containing media until 70-80% confluent. The media was then removed and replaced with serum-free media with PI. The cells were again incubated for 18-24 hours in order for the soluble L1 ectodomain and the L1-containing exosomes to accumulate in the media. The media was collected, filtered through a 0.2 micron filter to remove cell debris. The filtered media was then put through 3 rounds of centrifugation: 1,200 × g for 10 minutes, 10,000 × g for 20 minutes and 100,000 × g for 22 hours. The first two rounds of centrifugation were done using the Sorvall RC-5B centrifuge with a SS-34 (fixed angle) rotor mainly to further remove cell debris. The last round of centrifugation was done at 4°C using Beckman L8-55M Ultracentrifuge with a SW41 Ti (swinging bucket) rotor. Following the ultracentrifugation, the supernatant was removed and the glassy pellet that formed at the bottom of the ultracentrifuge tubes, which was presumed to contain exosomes, was resuspended in a small volume of sterile PBS (40-100 μl) with PI. The exosomal samples were analyzed by western blotting.

### Scratch assay and time-lapse microscopy

Motility of 9L/LacZ cells infected with retroviral vectors and incubated with anti-L1 antibodies was measured by using time-lapse microscopy as previously described [62,63]. Cells were grown to confluence either on 35 mm glass bottomed dishes (MatTek Corp., Ashland, MA or World Precision Instruments, Inc., Sarasota, FL) for 9L/LacZ infected with mouse antisense-L1 and control retroviral vectors or on plastic tissue culture dishes for 9L/LacZ cells incubated with anti-L1 antibodies. Monolayers were "wounded" by introducing scratches with a sterile plastic 1 ml pipettor tip in serum-free media. For antibody blocking experiments, ASCS4 and EZ1 antibodies were added as purified IgG to a concentration of 5 μg/ml and 40 μg/ml, respectively and the RGD-containing L1 peptide was added at 40 μg/ml. Cultures were then placed into a custom culture chamber mounted on a ProScan II automated stage (Prior Scientific, Rockland, MA) on a Nikon TE-2000E microscope. Temperature was maintained at 37°C by a combination of a warm air temperature controller (Air Therm, World Precision Instruments, Sarasota, FL) and thermoelectric warming with an optically clear temperature-controlled stage insert (Tokai Hit, Shizuoka-ken, Japan). The atmosphere within the chamber was kept at an average of 5% CO2/95% air using a gas injection controller (Forma Scientific, Marietta, OH). A CoolSnap ES CCD camera (Photometrics, Tucson, AZ) was used to capture images over the course of the experiment using a Nikon Plan Fluor 20× ELWD objective at areas of interest on each plate for approximately 20 h. Retroviral vector experiments entailed collection of phase contrast and fluorescence images (488 nm illumination) at 15 min intervals and antibody blocking experiments entailed collection of phase contrast images at 5 min intervals. The system was controlled using MetaMorph Premier Software (Molecular Devices Corporation, Downingtown, PA). Quantitative analysis of cell motility was performed on acquired sequential phase contrast images using the MetaMorph software "Track Points" feature with nucleoli serving as imaging targets. Data were evaluated statistically using Student's two-tailed t-test and graphed using Microsoft Excel.

## Abbreviations

ADAM10: A Disintegrin and Metalloprotease 10; FACS: Fluorescence-activated cell sorting or flow cytometry; FN Repeats: Fibronectin-like Repeats; GBM: Glioblastoma Multiforme; GFAP: Glial Fibrillary Acidic Protein; Ig Domains: Immunoglobulin-like Domains; NF-M: Neurofilament-M; RGD: Arg-Gly-Asp; RIPA buffer: Radioimmunoprecipitation Assay buffer; RT-PCR: Reverse Transcription-Polymerase Chain Reaction.

## Competing interests

The authors declare that they have no competing interests.

## Authors' contributions

All authors read and approved the manuscript. MY, SA, VP, MT, OB, EL, and DSG designed experiments, conducted experiments and analyzed data. MY and DSG drafted and edited the manuscript. JT carried out experiments and analyzed data. MB collected surgical glioma specimens following approved institutional protocols.
